# Frequent genetic aberrations in the cell cycle related genes in mucosal melanoma indicate the potential for targeted therapy

**DOI:** 10.1186/s12967-019-1987-z

**Published:** 2019-07-29

**Authors:** Longwen Xu, Zhiyuan Cheng, Chuanliang Cui, Xiaowen Wu, Huan Yu, Jun Guo, Yan Kong

**Affiliations:** Key Laboratory of Carcinogenesis and Translational Research (Ministry of Education), Department of Renal Cancer and Melanoma, Peking University Cancer Hospital & Institute, 52 Fucheng Road, Beijing, 100142 China

**Keywords:** Mucosal melanoma, Targeted therapy, *CDK4*, *CCND1*, *P16*^*INK4a*^

## Abstract

**Background:**

Melanoma is one of the most aggressive cancers with extremely poor prognosis, and the median survival time for stage IV patients is approximately 6 to 8 months. Unlike cutaneous melanoma, mucosal melanoma is a rare melanoma subtype among Caucasian patients but its incidence remains as high as 22.6% among Chinese patients. Screening specific genetic variations is the guideline to select targeted drugs for the treatment of advanced melanoma, whereas the genetic variation spectrum and potential therapeutic targets for mucosal melanoma are largely unclear. It is urgent to identify promising genetic variants for mucosal melanoma so as to develop effective targeted therapies for this disease.

**Methods:**

Tumor samples from 213 Chinese mucosal melanoma patients were involved in this study. P16^INK4a^/Cyclin D1/CDK4 copy number was examined using the QuantiGene Plex DNA assay and the correlation between abnormal copy number and clinicopathological parameters was analyzed. Patient-derived xenograft models (PDX) were performed to detect the effects of CDK4/6 inhibitors on the proliferation of mucosal melanoma cells with altered copy number of CDK4 pathway (*CDK4, Cyclin D1* and *P16*^*INK4*a^). The molecular mechanisms of CDK4/6 inhibitors on the proliferation of mucosal melanoma were analyzed by RNAseq.

**Results:**

Among the 213 samples, the amplification rate of *CDK4* and *CCND1* was 47.0% and 27.7%, respectively, and the deletion rate of *P16*^*INK4a*^ was 57.7%. Patients with more than one genetic abnormality were up to 81.7%. CDK4 pathway gene copy number variation was not associated with the prognosis of patients with mucosal melanoma (P > 0.05). Drug sensitivity tests showed that AT7519, a broad-spectrum CDK inhibitor, and PD0332991, a specific CDK4/6 inhibitor, exhibited higher inhibitory effect on CDK4 signaling pathway abnormal mucosal melanoma cells-derived PDX tumors growth than CDK4 signaling pathway normal ones. RNA-seq analysis showed that CDK4 inhibitors may affect tumor proliferation through multiple signaling pathways.

**Conclusions:**

Abnormal copy number of cell cycle related genes is frequently found in mucosal melanoma. CDK4/6 inhibitors significantly suppress the PDX tumor growth with abnormal CDK4 pathway. CDK4 signaling variations predict the effectiveness of CDK4 inhibitors in mucosal melanoma.

**Electronic supplementary material:**

The online version of this article (10.1186/s12967-019-1987-z) contains supplementary material, which is available to authorized users.

## Background

Melanoma is one of the most aggressive cancers with extremely poor prognosis. Approximately 100,000 new cases of cutaneous melanoma (CM) are diagnosed and 7000 death occurs in the US in 2019 [1]. In China, the incidence of malignant melanoma is gradually growing, with an average of more than 20,000 new cases [2]. Because of the increased number of melanoma patients, more attention has been paid to the prevention and treatment of melanoma in China. Mucosal melanoma (MM), an aggressive subtype of melanoma, is extremely rare among Caucasian melanoma patients, with the incidence rate less than 2% [3]. However, the incidence of MM is very high, accounting for 22.6% among Chinese melanoma patients [4]. Compared with other subtypes, MM is characterized by occult location, late stage of initial diagnosis, high recurrence and metastasis rate. Due to the difficulty of diagnosis and treatment, the prognosis of MM is significantly poor, with a 14% 5-year survival. For Stage IV patients, the average survival time was only 6–8 months, and the 5-year survival rate was less than 5% [4, 5]. It is important to develop promising diagnostic biomarkers and effective treatment strategies for MM.

Genomic analysis shows that the major genetic variation of MM is the large-scale structural amplification or deletion of chromatin, distinct from the point mutation hotspot in CM [6–9]. Therefore, MM is different from CM in its pathogenesis, diagnosis, treatment and response to drugs. For instance, BRAF active mutation is common (> 50%) in CM, whereas the mutation rate is only about 10% in MM [6, 10]. Moreover, the effect of such targeted therapy on the treatment for MM was unclear due to the limited numbers of MM patients included in clinical trials. Copy number aberrations study may be helpful to investigate the pathogenesis of MM and to screen effective drug targets.

Abnormal cell cycle progression, caused by the mutation or amplification of CDK or cell cycle families, is one of the hallmarks of tumor cells [11]. CDKN2A/CDK4/6/CCND1 complex is the essential regulator of cell cycle. CDK4/6/CCND1 complex phosphorylates the retinoblastoma (Rb) and inhibits its activity, leading to the transition of G1 to S phase. By contrast, CDKN2A negatively regulates the progression of cell cycle. The combination of Palbociclib (a specific inhibitor of CDK4/6) and MLN0128 (an inhibitor of mTOR) significantly inhibits the proliferation of patient derived xenograft (PDX) model of ER-positive breast cancer [12]. High throughput sequencing in nasopharyngeal carcinoma reveals an increase in CCND1 copy number combined with CDKN2A gene deletion. Palbociclib significantly inhibits CDK4 signaling pathway activation in patient derived xenograft (PDX) model [13]. Palbociclib has antitumor activity for NRAS-mutant melanomas in a preclinical mouse model, indicating the CDK4 pathway as a potential therapeutic target [14]. A large cohort analysis in 2017 found that CDK4 gene amplification was higher in acral melanoma (AM), suggesting that CDK4 may be a therapeutic target for AM [15–17].

It is not clear whether there are variations in cell cycle related signaling pathways in MM and whether they can be used as effective therapeutic targets. To address this question, we collected 213 MM samples and investigated the status of CDK4 signaling related genes (CDK4, CCND1 and CDKN2A) and its relationship with clinical pathologic parameters. We also explored the inhibitory effects of CDK4/6 inhibitors on PDX tumor growth containing normal or aberrant CDK4 pathway. The results of this study may provide a new idea for the clinical treatment of MM.

## Materials and methods

### Patients and tissue samples

This study involved samples from primary lesions of 213 MM patients, who were hospitalized during January 2007 and October 2018 at the Peking Cancer Hospital & Institute. We obtained all the clinical and pathological data by medical record review, including age, gender, ulceration, depth of invasion, anatomic site, treatment, tumor-node-metastases stage, mutation status of therapeutic targets (such as KIT, BRAF and NRAS), follow-up time and survival (follow-up persisted until June 2018, or until the missing of follow-up or death of patients). An informed consent was obtained from all participants that were enrolled in clinical trials. This study was approved by the Medical Ethics Committee of the Beijing Cancer Hospital & Institute and was conducted according to the Declaration of Helsinki Principles.

### QuantiGenePlex DNA assay

Tissue homogenates were prepared according to the protocols in the user manual of QuantiGene Sample Processing Kit for Formalin-Fixed, Paraffin-Embedded Tissues (FFPE; Panomics of Affymetrics, Santa Clara, CA). Briefly, 5 to 8 pieces of deparaffinized sections (4 to 10 μm) were incubated with 150 μl homogenizing solution supplemented with 1.5 μl of proteinase K (50 μg/μl) at 65 °C for 6 h. The tissue homogenates were separated from debris by brief centrifugation and transferred to a new tube.

The branched DNA (bDNA) assay was performed according to the procedures described in the user manual of QuantiGenePlex DNA Assay (Panomics). Briefly, the homogenate DNA was sheared by the Covaris S2 (Covaris, Woburn, MA) with the following settings: duty cycle 5%, intensity 3, cycles/burst 200, 80 s. For each assay well, 40 μl homogenate was denatured with 2.5 M NaOH (final concentration 0.18 M) in the presence of DNA probe. Neutralized tissue homogenate was transferred to each well of the Hybridization Plate containing Working Bead Mix. All the samples were in duplicates. Hybridization Plate was sealed and incubated at 54 °C ± 1 °C in a shaking incubator (600 rpm) for 18–22 h. The unbound samples were washed away using the Bio-plex pro II wash station (Bio-Rad, Hercules, CA). Then the beads were sequentially hybridized with the DNA Pre-Amplifier, DNA Amplifier, Label Probe and SAPE (Streptavidin-conjugated R-phycoerythrin). Fluorescence intensities were measured by the Bio-plex 100 system (Bio-Rad).

The mean fluorescence intensities of the duplicates were calculated for all genes. The background values were subtracted from each probe set signal. Values of tested genes were normalized to the geometric means of *Rpph1*, *Rpp30* and *Rplp0*. For each test sample, normalized signal was divided by the reference DNA sample (G1521, Promega, Madison, WI) for each test gene, and the values were multiplied by the known copy number (usually 2 copies) of each gene in the reference genome. The *Cdk4*, *Ccnd1* and *P16*^*INK4a*^ copy numbers for the samples were calculated by dividing the sample values tothe control values: No gain referred to copy number ≤ 2, gain referred to copy number > 2, loss referred to copy number < 1.

### DNA preparation and TaqMan copy number assays

Genomic DNA was extracted from FFPE sections using a QIAamp DNA FFPE Tissue Kit (Qiagen, Hilden, Germany). To validate the results of QuantiGenePlex DNA Assay, the copy numbers of Cdk4, Ccnd1 and P16^INK4a^ were further quantified by TaqMan Copy Number Assays (Applied Biosystems of ThermoFisher, Waltham, MA). A TaqMan probe targeted on the Rnasep gene was used as a control. Quantitative real-time PCR was performed using the ABI 7500 FAST real-time PCR system (Applied Biosystems). Copy numbers were then determined by CopyCaller v2.0 software (Applied Biosystems) using the comparative Ct (ΔΔCt) method.

### Immunohistochemistry assay

Immunohistochemistry analyses were performed using antibodies against Ki67 (dilution 1:400) (Abcam, Cambridge, UK) as described (11, 17). The Ki-67+ cells under 5 random fields were counted and were presented as mean ± SD of three sections.

### Cell culture

The HMVII cell line was obtained from Sigma-Aldirch and was cultured at 37 °C in Ham’s F-10 medium supplemented with 1% penicillin and streptomycin (Invitrogen) and 10% fetal bovine serum (HyClone of GE Healthcare, Logan, UT). The GAK cell line was obtained from JCRB Cell Bank and was cultured at 37 °C in Ham’s F-12 medium supplemented with 1% penicillin and streptomycin (Invitrogen) and 10% fetal bovine serum (HyClone of GE Healthcare, Logan, UT).

### Cell proliferation assays

CDK4/6 inhibitors including PD0332991 (#S1116), and pan-CDK inhibitor AT7519 (#S1524) were purchased from Selleck Chemicals (Houston, TX). All inhibitors were dissolved at 10 mM in dimethylsulfoxide (DMSO) as stock solutions. After treatment with various concentrations of inhibitors or DMSO for 24 h, proliferation of the cells was evaluated using the Cell Titer-Glo Luminescent Cell Viability Assay (Promega) according to the instructions.

### Patient-derived xenograft (PDX) model and treatment

Fragments of patient-derived MM tissues bearing typical CDK4 pathway aberrations were cut into fragments and then subcutaneously inoculated into a 6 week-old NOD/SCID (non-obese diabetic and severe combined immunodeficiency) female mouse (4–6 week-old, 18–22 g-weight) to establish the PDX model. The established PDX model was called passage 0 (P0). When the tumor size reached approximately 500 mm^3^, the mice were sacrificed, and tumor tissues were separated and re-inoculated into new mice to obtain the subsequent passages called P1, P2, P3 and forth. 10 PDX models containing typical CDK4 pathway aberrations (Table 1) were finally established.

**Table 1:** The basic information of 10 PDX models

Code	Gender	Age	Ulceration	Anatomic site	Stage	*CDK4*	*CCND1*	*P16*^*INK4a*^	*KIT*	*BRAF*	*NRAS*
PDX-001	M	49	Yes	1	IV	Normal	Normal	Normal	WT	WT	WT
PDX-002	M	57	Yes	4	IV	Normal	Normal	Normal	W557R	WT	WT
PDX-003	F	78	Yes	5	II	Gain	Gain	Loss	WT	WT	WT
PDX-004	F	43	Yes	5	III	Gain	Gain	Loss	L576P	WT	WT
PDX-005	F	57	Yes	5	II	Gain	Gain	Loss	WT	WT	WT
PDX-006	M	58	Yes	3	I	Gain	Gain	Loss	WT	WT	WT
PDX-007	F	71	Yes	2	II	Gain	Gain	Normal	WT	WT	WT
PDX-008	F	65	No	1	II	Gain	Gain	Normal	WT	WT	G12C
PDX-009	F	69	Yes	1	II	Normal	Gain	Loss	WT	WT	Q61L
PDX-010	M	55	Yes	2	II	Normal	Gain	Normal	WT	WT	WT

*F* female, *M* male, *WT* wild type

a: Anatomic site: 1 = nasopharynx; 2 = oral cavities; 3 = esophagus; 4 = anorectum; 5 = genitourinary

Mice (P2) were randomized (treatment arm versus control arm; n = 4) and treated with control (sodium lactate buffer, pH 4.5) or PD0332991 and with control (saline solution, pH 4.0) or AT7519. For PD0332991 treatment, mice received PD0332991 (50 mg/kg in pH 4.5 sodium lactate buffer) via oral gavage daily. For AT7519 treatment, mice received AT7519 (12 mg/kg in saline solution) via intraperitoneal injection daily. Tumor sizes were measured every 3 days and tumor volume was calculated using the formula: volume = length*width^2^/2. Percentage of tumor volume on day of treatment (baseline volume) was used as the end-point of study. The treatment lasted for 14 days, after which the mice were sacrificed and the tumors were fixed in 10% formalin for histological and immunohistological analysis. All animal care and experimental procedures were performed in consistent with the Animal Care Ethics approved by the Medical Ethics Committee of the Beijing Cancer Hospital & Institute.

### RNA-sequencing

RNA-sequencing was performed at the Shanghai Biotechnology Company. cDNA library was built according to the standard manufacturer’s protocol. Paired-end 2 × 100 bp read sequencing was performed using the Illumina HiSeq X-ten (Illumina, USA). The FASTX-Toolkit (v0.0.13) was used to trim low-quality bases. High-quality reads were aligned to the human GRCh38 reference genome with two mismatches using spliced mapping alignment in Hisat2 (version: 2.0.4). After genome mapping, Stringtie (version: 1.3.0) was run with a reference annotation to generate FPKM values for known gene models. Differentially expressed genes were identified using edgeR. The *P* value significance threshold in multiple tests was set by the false discovery rate (FDR). The fold-changes were also estimated according to the FPKM (Fragments Per Kilobase of exon model per Million mapped read) in each sample. The differentially expressed genes were selected using the following filter criteria: FDR ≤ 0.05 and fold-change ≥ 2.

### Statistical analysis

Statistical analyses were performed using SPSS 22.0 software. Continuous data such as age was described using mean ± SD for normally distributed data. The correlations between aberration status and clinical parameters were evaluated by Chi square test or Fisher’s exact test. Kaplan–Meier estimates of time-to-event overall survival (OS) and follow-up time were calculated. Log-rank tests were used to estimate the statistical significance between the time-dependent outcomes of OS. All statistical analyses were two-sided, and *P *< 0.05 was considered as statistically significant.

## Results

### Aberrations of Cdk4, Ccnd1 and P16^INK4a^ in MM

Firstly, we detected the copy number variation of CDK4, CCND1 and P16^INK4a^ genes in paraffin sections of 213 cases of MMs through QGP method (Table 2). Among the 213 samples, 100 cases (47.0%), 59 cases (27.7%) and 123 cases (57.7%) showed Cdk4 gain, Ccnd1 gain, and P16^INK4a^ loss, respectively. Moreover, 36.6% of MMs contained more than two concurrent aberrations, and 8.0% of MMs contained three aberrations. The overall frequency of MM containing any CNV (≥ 1 CNV) was 81.7%, whereas 39 cases harbored no CNV aberrations in these three genes. 38 cases had Ccnd1 loss, and one case harbored P16^INK4a^ gain (Table 2). Collectively, Cdk4 gain, Ccnd1 gain and/or P16^INK4a^ loss were observed in most MMs.

**Table 2:** Copy number variations of genes related to CDK4 pathway and mutation status of therapeutic targets in mucosal melanoma

CNV status	N = 213	Genetic mutation of therapeutic targets		
	N (%)	% (No. positive cases/no. examined cases)		
		*KIT*	*BRAF*	*NRAS*
≥ 1 CNV				
*CDK4* gain	100 (47.0)	7.4 (7/95)	5.2 (5/96)	15.7 (11/70)
2.5–5 copies	76 (35.7)	5.6 (4/71)	5.6 (4/72)	19.6 (11/56)
5–10 copies	14 (6.6)	11.8 (2/14)	0 (0/14)	0 (0/10)
>10 copies	10 (4.7)	10.0 (1/10)	10.0 (1/10)	0 (0/4)
*CCND1* gain	59 (27.7)	1.8 (1/56)	8.9 (5/56)	10.4 (5/48)
2.5–5 copies	49 (23.0)	0 (0/46)	10.9 (5/46)	112.8 (5/39)
5–10 copies	9 (4.2)	11.1 (1/9)	0 (0/9)	0 (0/8)
> 10 copies	1 (0.5)	0 (0/1)	0 (0/1)	0 (0/1)
*P16*^*INK4a*^ loss	123 (57.7)	9.6 (11/114)	4.3 (5/115)	10.5 (9/86)
Overall	174 (81.7)	7.0 (11/199)	10.0 (10/200)	13.7 (21/153)
≥ 2 CNVs				
*CDK4* gain plus*CCND1* gain	13 (6.1)	0 (0/12)	0 (0/12)	20.0 (2/10)
*CDK4* gain plus *P16*^*INK4a*^loss	49 (23.0)	12.8 (6/47)	4.2 (2/48)	12.1 (4/33)
*CCND1* gain plus *P16*^*INK4a*^loss	16 (7.5)	6.3 (1/16)	0 (0/16)	15.4 (2/13)
Overall	78 (36.6)	9.3 (7/75)	3.9 (3/76)	14.3 (8/56)
3 CNVs				
Overall	17 (8.0)	0 (0/16)	12.5 (2/16)	0 (0/14)

*CNV* copy number variation

### Correlation of CDK4 pathway aberrations to other driver genes

We also detected the mutation status of c-Kit (exon 9, 11, 13, 17 and 18), BRAF (exon 11 and 15) and NRAS (exon 1 and 2) by Sanger sequencing. The result showed that the mutation rate of c-Kit, BRAF and NRAS was 7.4%, 5.2% and 15.7% in MM patients carrying Cdk4 gain, respectively. For MM patients carrying Ccnd1 gain, the mutation rate of c-Kit, BRAF and NRAS was 1.8%, 8.9%, 10.4%, respectively. For MM patients carrying P16^INK4a^, the mutation rate of c-Kit, BRAF and NRAS was 9.6%, 4.3%, 10.5%, respectively (Table 2).

### Correlation of Anatomic site to TNM stage of mucosal melanoma

We next explored the anatomic sites of MM in our 213 MM patient’s cohort. The prevalent anatomic sites were head and neck (44.2%), anorectum (21.1%), genitourinary (25.8%) and oesophagus (8.9%) (Table 4). The proportion of patients with TNM stage I, II, III, and IV diseases were 2.8%, 53.5%, 28.6% and 15.1%, respectively. The percentages of patients with stage IV of MM from head and neck were significantly lesser than other anatomic sites (P < 0.001).

### Correlation of CDK4 pathway aberrations to clinicopathological features

Last, we investigated the correlations between the aberration of Cdk4, Ccnd1 and P16^INK4a^ and the clinicopathological features of MM. Statistical analysis was divided into 8 groups: Cdk4 aberration, Ccnd1 aberration, P16^INK4a^ aberration, ≥ 1 CNV (copy number variation), Cdk4 gain + Ccnd1gain, Cdk4 gain + P16^INK4a^ loss, Ccnd1gain + P16^INK4a^ loss and Cdk4 gain + Ccnd1gain + P16^INK4a^ loss.

In our cohort, both age, gender, anatomic site and follow-up time were not significantly different between patients with or without any CNVs for Cdk4, Ccnd1, P16^INK4a^ or other indicated stochastic combinations. However, the other clinical features were at least significantly associated with a molecular variant of the CDK4 signaling pathway: compared with P16^INK4a^ loss, patients with normal were more likely to ulcerate;The depth of invasion of P16^INK4a^ normal group tended to be deeper than that of group with P16^INK4a^ loss, while there was no statistical difference (P = 0.053). The percentages of patients with stage I–IV of MM were significantly different between patients with CDK4 gain and CDK4 normal (P = 0.004; Table 3). The TNM stage of ≥ 1 CNV group tended to be greater than that of group without any CDK4 pathway aberrations, while there was no statistical difference (P = 0.059);Similar result was observed in the group of CDK4 gain + P16^INK4a^ loss (P = 0.088). The treatment groups for MM patients was significantly different between patients with CDK4 gain and CDK4 normal (P = 0.003; Additional file 1: Table S1).

**Table 3:** Correlation of CDK4 pathway aberrations to clinicopathologic features of mucosal melanoma

Clinicopathologic factor	CDK4 aberration			CCND1 aberration			
	Gain	Normal	P value	Gain	Loss	Normal	P value
Age (year)			0.552				0.853
Median (range)	56.5 ± 11.6	54.9 ± 11.3		56.4 ± 11.3	55.3 ± 11.6	55.4 ± 11.6	
Gender n (%)			0.409				0.413
Male	41 (41.0)	39 (35.5)		20 (33.9)	18 (47.4)	45 (38.8)	
Female	59 (59.0)	71 (64.5)		39 (66.1)	20 (52.6)	71 (61.2)	
Ulceration n (%)			0.555				0.769
Yes	52 (52.0)	49 (44.5)		25 (42.4)	20 (52.6)	58 (50.0)	
No	20 (20.0)	26 (23.6)		14 (23.7)	9 (23.7)	23 (19.8)	
NA	28 (28.0)	35 (31.8)		20 (33.9)	9 (23.7)	35 (30.2)	
Depth of invasion			0.981				0.853
T1 ≤ 1 mm	13 (13.0)	16 (14.5)		10 (16.9)	4 (10.5)	15 (12.9)	
T2 1–2 mm	21 (21.0)	24 (21.8)		10 (16.9)	11 (28.9)	25 (21.6)	
T3 2–4 mm	18 (18.0)	20 (18.2)		10 (16.9)	6 (15.8)	22 (19.0)	
T4 > 4 mm	48 (48.0)	50 (45.5)		29 (49.2)	17 (44.7)	54 (46.6)	
Anatomic site n (%)			0.153				0.885
Head and neck	51 (51.0)	41 (37.3)		27 (45.8)	15 (39.5)	52 (44.8)	
Oesophagus	8 (8.0)	11 (10.0)		3 (5.1)	5 (13.2)	11 (9.5)	
Anorectum	21 (21.0)	23 (20.9)		13 (22.0)	9 (23.7)	23 (19.8)	
Genitourinary	20 (20.0)	35 (31.8)		16 (27.1)	9 (23.7)	30 (25.9)	
Stages n (%)			*0.004*				0.239
I	5 (5.0)	1 (0.9)		0 (0.0)	1 (2.6)	5 (4.3)	
II	46 (46.0)	67 (60.9)		30 (50.8)	26 (68.4)	58 (50.0)	
III	38 (38.0)	22 (20.0)		18 (30.5)	9 (23.7)	34 (29.3)	
IV	11 (11.0)	20 (18.0)		11 (18.6)	2 (5.3)	19 (16.4)	
Survival (months)			0.054				0.702
Median (95% CI)	45.0 (39.3, 50.7)	41.2 (35.4, 47.0)		43 (40.2, 45.8)	47.4 (38.9, 55.9)	45.0 (40.0, 50.0)	
Median follow-up time (95% CI)	38.1 (26.0, 50.2)	37.0 (25.5, 48.5)	0.322	44.5 (27.4, 61.6)	39.5 (22.2, 56.8)	31.0 (18.3, 43.7)	0.453

Clinicopathologic factor	P16^INK4a^ aberration			Overall aberration (≥ 1 CNV)		
	Loss	Normal	P value	Yes	No	P value
Age (year)			0.733			0.581
Median (range)	55.4 ± 11.5	56.2 ± 11.4		55.5 ± 11.4	56.3 ± 12.0	
Gender n (%)			0.903			0.771
Male	48 (39.0)	34 (38.2)		67 (38.5)	16 (41.0)	
Female	75 (61.0)	55 (61.8)		107 (61.5)	23 (59.0)	
Ulceration n (%)			*0.014*			0.189
Yes	52 (42.3)	51 (57.3)		81 (46.6)	22 (56.4)	
No	25 (20.3)	21 (23.6)		36 (20.7)	10 (25.6)	
NA	46 (37.4)	17 (119.1)		36 (20.7)	7 (17.9)	
Depth of invasion			0.053			0.348
T1 ≤ 1 mm	20 (16.3)	9 (10.1)		26 (14.9)	3 (7.7)	
T2 1–2 mm	33 (26.8)	13 (14.6)		40 (23.0)	6 (15.4)	
T3 2–4 mm	19 (15.4)	19 (21.3)		29 (16.7)	9 (23.1)	
T4 > 4 mm	51 (41.5)	48 (53.9)		79 (45.4)	21 (53.8)	
Anatomic site n (%)			0.379			0.691
Head and neck	55 (44.7)	39 (43.8)		79 (45.4)	15 (38.5)	
Oesophagus	14 (11.4)	5 (5.6)		16 (9.2)	3 (7.7)	
Anorectum	22 (17.9)	22 (24.7)		34 (19.5)	11 (28.2)	
Genitourinary	32 (26.0)	23 (25.8)		45 (25.9)	10 (25.6)	
Stages n (%)			0.515			
I	4 (3.3)	2 (2.2)		5 (2.9)	1 (2.6)	0.059
II	65 (52.8)	49 (55.1)		90 (51.7)	24 (61.5)	
III	39 (31.7)	22 (24.7)		56 (32.2)	5 (12.8)	
IV	15 (12.2)	16 (18.0)		23 (13.2)	9 (23.1)	
Survival (months)			0.528			0.169
Median (95% CI)	43.6 (40.0, 47.2)	43.6 (32.3, 54.9)		44.0 (40.4, 47.6)	43.6 (18.6, 68.6)	
Median follow-up time (95% CI)	39.7 (29.7, 49.7)	33.7 (22.3, 45.1)	0.137	38.1 (31.7, 44.5)	21.9 (17.8, 26.0)	0.206

Clinicopathologic factor	CDK4 gain + CCND1 gain			CDK4 gain + P16^INK4a^ loss		
	Positive	Negative	P value	Positive	Negative	P value
Age (year)			0.866			0.722
Median (range)	57.6 ± 11.1	55.4 ± 11.5		56.2 ± 11.1	55.5 ± 11.6	
Gender n (%)			0.817			0.382
Male	12 (40.0)	68 (37.8)		28 (42.4)	52 (36.1)	
Female	18 (60.0)	112 (62.2)		38 (57.6)	92 (63.9)	
Ulceration n (%)			0.690			0.971
Yes	13 (43.3)	88 (48.9)		31 (47.0)	70 (48.6)	
No	6 (20.0)	40 (22.2)		15 (22.7)	31 (21.5)	
NA	11 (36.7)	52 (28.9)		20 (30.3)	43 (29.9)	
Depth of invasion			0.767			0.527
T1 ≤ 1 mm	3 (10.0)	26 (14.4)		8 (12.1)	21 (14.6)	
T2 1–2 mm	5 (16.7)	40 (22.2)		18 (27.3)	27 (18.8)	
T3 2–4 mm	6 (20.0)	32 (17.8)		10 (15.2)	28 (19.4)	
T4 > 4 mm	16 (53.3)	82 (45.6)		30 (45.5)	68 (47.2)	
Anatomic site n (%)			0.160			0.935
Head and neck	16 (53.3)	76 (42.2)		31 (47.0)	61 (42.4)	
Oesophagus	3 (10.0)	16 (8.9)		6 (9.1)	13 (9.0)	
Anorectum	8 (26.7)	36 (20.0)		13 (19.7)	31 (21.5)	
Genitourinary	3 (10.0)	52 (28.9)		16 (24.2)	39 (27.1)	
Stages n (%)			0.213			0.088
I	0 (0.0)	6 (3.3)		4 (6.1)	2 (1.4)	
II	12 (40.0)	101 (56.1)		32 (48.5)	81 (56.2)	
III	12 (40.0)	48 (26.7)		23 (34.8)	37 (25.7)	
IV	6 (20.0)	25 (13.9)		7 (10.6)	24 (16.7)	
Survival (months)			0.757			0.174
Median (95% CI)	44.0 (41.4, 46.6)	43.6 (39.3, 47.9)		44.0 (41.4, 46.6)	43.0 (37.6, 48.4)	
Median follow-up time (95% CI)	54.0 (29.2, 78.8)	37.0 (30.0, 44.0)	0.636	38.1 (25.9, 50.3)	37.0 (29.1, 44.9)	0.153

Clinicopathologic factor	P16^INK4a^ loss + CCND1 gain			CDK4 gain + P16^INK4a^ loss + CCND1 gain		
	Positive	Negative	P value	Positive	Negative	P value
Age (year)			0.531			0.807
Median (range)	58.1 ± 12.5	55.3 ± 11.2		59.2 ± 12.8	55.3 ± 11.4	
Gender n (%)			0.927			0.476
Male	13 (39.4)	69 (38.5)		8 (47.1)	75 (38.3)	
Female	20 (60.6)	110 (61.5)		9 (52.9)	121 (61.7)	
Ulceration n (%)			0.220			0.857
Yes	13 (39.4)	90 (50.3)		8 (47.1)	95 (48.5)	
No	6 (18.2)	40 (22.3)		3 (17.6)	43 (21.9)	
NA	14 (42.4)	49 (27.4)		6 (35.3)	58 (29.6)	
Depth of invasion			0.584			0.682
T1 ≤ 1 mm	7 (21.2)	22 (12.3)		1 (5.9)	28 (14.3)	
T2 1–2 mm	7 (21.1)	39 (21.8)		3 (17.6)	43 (21.9)	
T3 2–4 mm	5 (15.2)	33 (18.4)		3 (17.6)	35 (17.9)	
T4 > 4 mm	14 (42.4)	85 (47.5)		10 (58.8)	90 (45.9)	
Anatomic site n (%)			0.404			0.497
Head and neck	18 (54.5)	76 (42.5)		9 (52.9.6)	85 (43.4)	
Oesophagus	1 (3.0)	18 (10.0)		1 (5.9)	18 (9.2)	
Anorectum	6 (18.2)	38 (21.1)		5 (29.4)	40 (20.4)	
Genitourinary	8 (24.2)	47 (26.1)		2 (11.8)	53 (27.0)	
Stages n (%)			0.383			0.268
I	0 (0.0)	6 (3.4)		0 (0.0)	6 (3.1)	
II	16 (48.5)	98 (54.7)		6 (35.3)	108 (55.1)	
III	13 (39.4)	48 (26.8)		8 (47.1)	53 (27.0)	
IV	4 (12.1)	27 (15.1)		3 (17.6)	29 (14.8)	
Survival (months)			0.287			0.145
Median (95% CI)	42 (35.0, 49.0)	45.5 (40.8, 50.2)		42 (33.5, 50.5)	45 (40.5, 49.4)	
Median follow-up time (95% CI)	55.5 (20.3, 90.7)	35.0 (27.6, 42.4)	0.102	38.1 (25.9, 50.3)	37.0 (29.1, 44.9)	0.153

The overall survival of MM patients with Cdk4 gain, Ccnd1 gain, P16^INK4a^ loss or other indicated stochastic combinations were comparable. Univariate Cox analysis suggested thatCdk4 gain, Ccnd1 gain, P16^INK4a^ loss or other stochastic combinations might not be of prognostic significance for melanoma patients (Table 3).

### Sensitivity of MM cells to CDK4/6 inhibitors

**Fig. 1:**
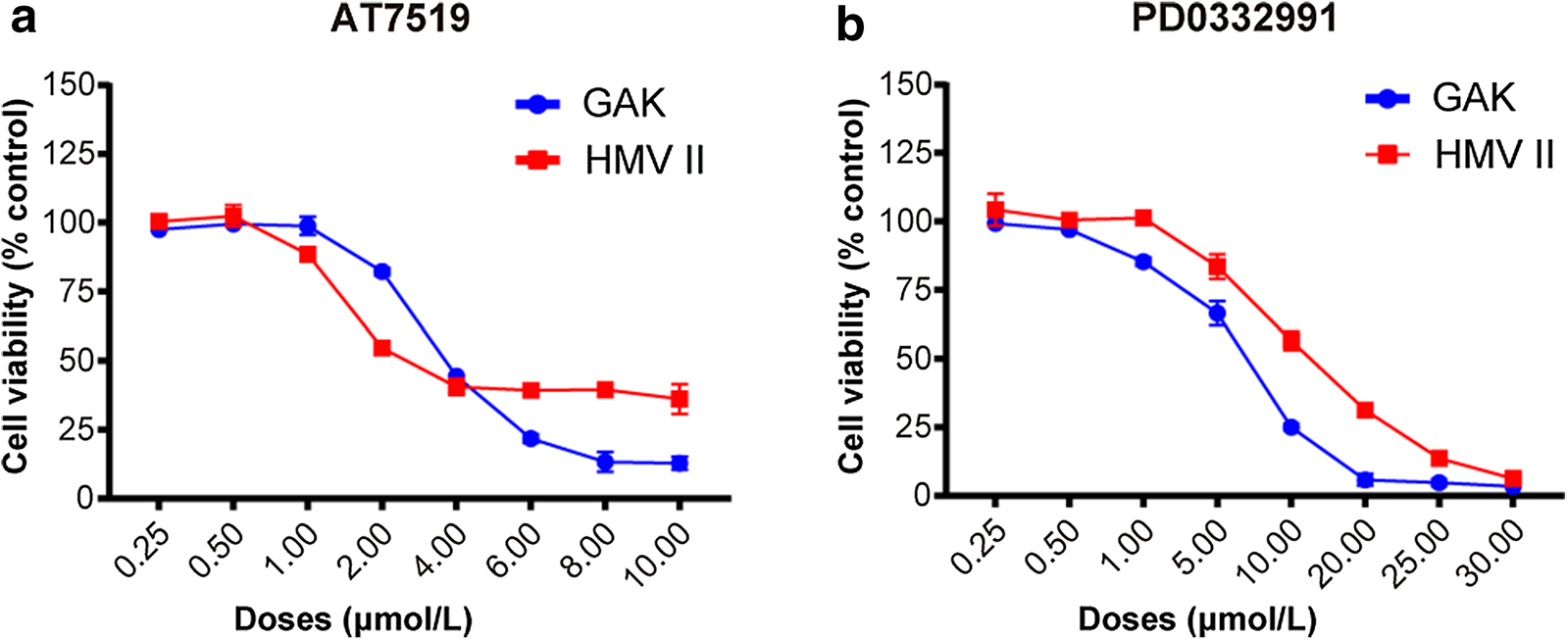
Sensitivity of mucosal melanoma cells to CDK4/6 inhibitors. The proliferation was evaluated by Cell Titer-Glo method (**a**, **b**), and the results were presented as mean ± SD of three independent experiments. The statistical significance of the growth curves was evaluated by repeated measure variance analysis

To evaluate the effect of CDK4/6 inhibition on the proliferation of MM cells, GAK and HMV II cell lines were treated with different concentrations of pan-CDK inhibitor AT7519 and CDK4/6 specific inhibitor PD0332991. The genetic variations of key genes for these cells are listed in Table 4. Both pan-CDK inhibitor AT7519 and CDK4/6 specific inhibitor PD0332991 significantly inhibited the viability of GAK and HMV II cells. For AT7519, the inhibitory rate in GAK and HMV II cells was obvious when higher than 4 µ mol/l. For PD0332991, GAK and HMV II were strikingly sensitive at a concentration higher than 10 µ mol/l (Fig. 1).

**Table 4:** Correlation of Anatomic sites to TNM stage of mucosal melanoma

Anatomic site	Patients (no. %)	TNM分期 (no.%)				P-value
		I	II	III	IV	< 0.001
Head and neck	94 (44.2)	3 (3.2)	57 (60.6)	31 (33.0)	3 (3.2)	
Nasopharynx	60 (28.2)	1 (1.7)	48 (80.0)	10 (16.7)	1 (1.7)	
Oral cavity	34 (16.0)	2 (5.9)	9 (26.5)	21 (61.8)	2 (5.9)	
Oesophagus	19 (8.9)	1 (5.3)	6 (31.6)	8 (42.1)	4 (21.1)	
Anorectum	45 (21.1)	1 (2.2)	15 (33.3)	14 (31.1)	15 (33.3)	
Genitourinary^a^	55 (25.8)	1 (1.8)	36 (65.5)	8 (14.5)	10 (18.2)	
Total	213	6 (2.8)	114 (53.5)	61 (28.6)	32 (15.1)	

^a^The sites of genitourinary mucosal melanomas included vulva, vagina, urethra and cervix

### Sensitivity of PDX models to CDK4/6 inhibitors

To analyze the sensitivity of MM containing typical CDK4 pathway aberrations to CDK4/6 inhibitors, we selected 10 PDX models with 5 different CDK4 pathway aberrations. The basic information of the PDX models is shown in Table 1. Two models (PDX-001 and PDX-002) are CDK4 pathway normal. Four models (PDX-003, PDX-004, PDX-005 and PDX-006) are *Cdk4* gain + *Ccnd1* gain + *P16*^*INK4a*^ loss. Two models (PDX-007 and PDX-008) are *Cdk4* gain + *Ccnd1* gain. One model (PDX-009) is *Ccnd1* gain + *P16*^*INK4a*^ loss. One model (PDX-010) is *Ccnd1* gain.

**Fig. 2:**
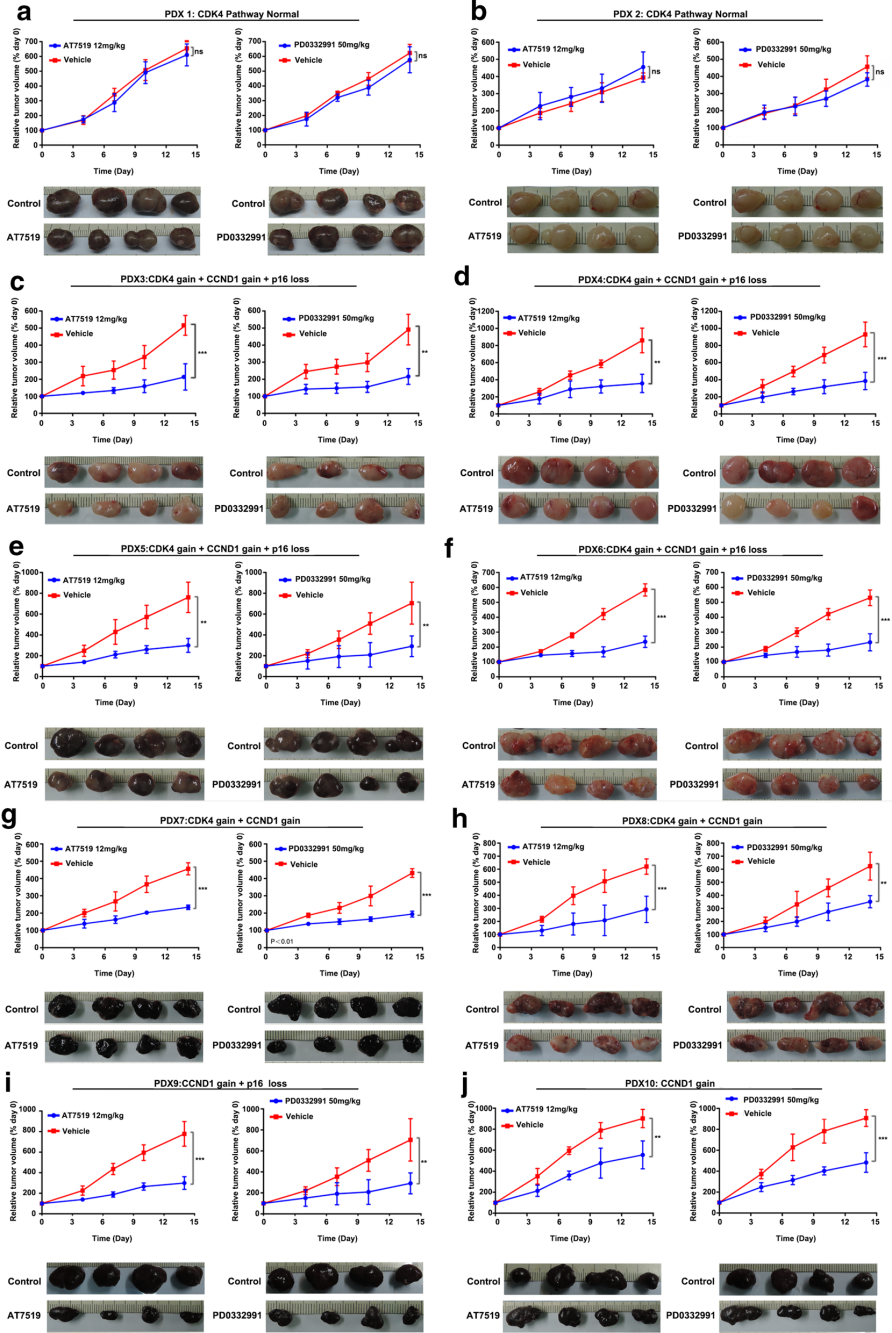
Sensitivity of PDX models containing CDK4 aberrations to CDK4/6 inhibitors in vivo. When the tumor size reached approximately 600 mm^3^, mice (n = 4 per group) were treated with buffer control or inhibitors daily. Tumor volume was evaluated as % of the tumor volume on day 0 and presented as mean ± SD. The comparison of the growth curves was done with the repeated measure variance analysis. *ns* no significances; **P < 0.01; ***P < 0.001

**Fig. 3:**
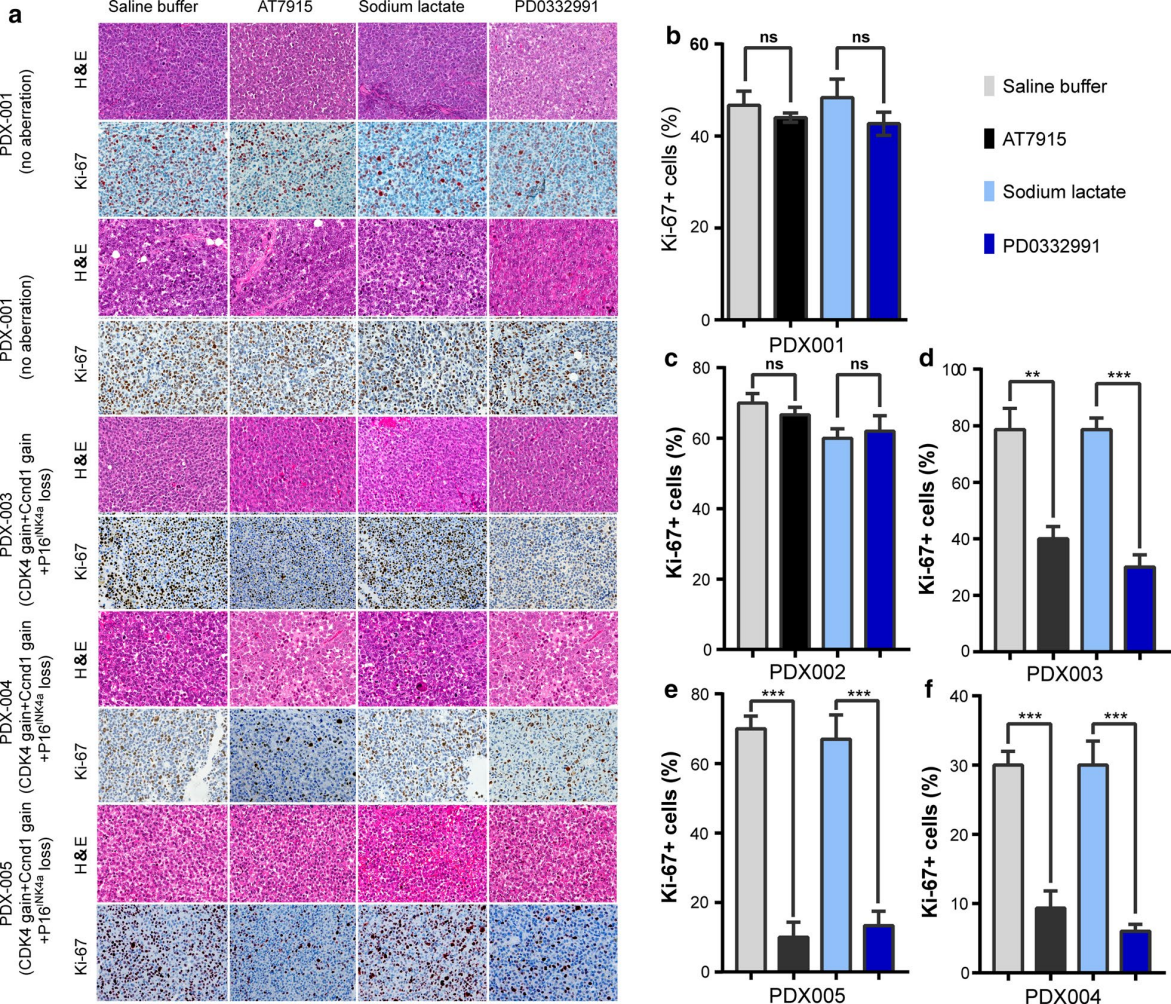

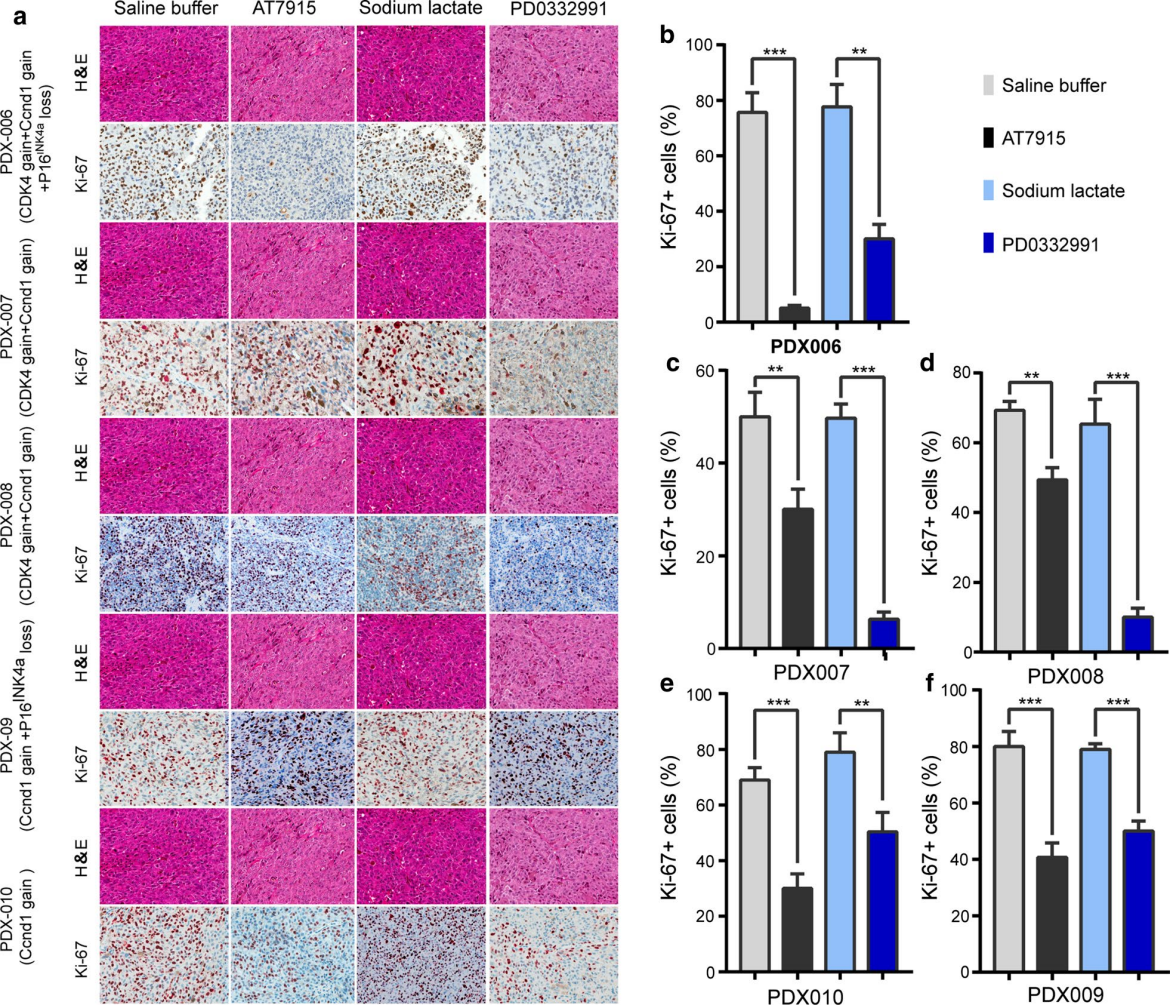
Proliferation index of mucosal melanoma cells from PDX models containing CDK4 aberrations after CDK4/6 inhibitors treatments. On day 14 of treatments, the tumor nodules were excised and examined by H&E staining and immunohistochemical staining (for Ki-67). The sections were evaluated under microscope, and typical staining was photographed (**a**). The Ki-67 + cells under 5 random fields were counted. Bar = 20 μm. The results of Ki-67 + cells (**b**–**f**) were presented as mean ± SD of three sections. *ns* no significances; *P < 0.05; **P < 0.01; ***P < 0.001

Then we treated the PDX models with AT7519 and PD0332991. As compared to the vehicle-treated group, AT7519 and PD0332991 showed no inhibitory effect on tumor growth in PDX-001 and PDX-002 model. Interestingly, AT7519 and PD0332991 significantly retarded the tumor growth of PDX-003 to PDX-010 (Fig. 2). To confirm our findings, we performed immunohistochemical staining of Ki-67 in these tumors. Consistently, the number of Ki-67+ cells was significantly decreased after AT7519 and PD0332991 treatments in PDX models with CDK4 pathway aberration (Fig. 3). Taken together, these data indicate that CDK4 aberration dictates the sensitivity of MM-derived PDX tumor growth to CDK inhibitors (Table 5).

**Table 5:** The mutation status of GAK and HMV II

Cells	*CDK4*	*CCND1*	*P16*^*INK4a*^	*BRAF*^*a*^	*NRAS*^*a*^	*CKIT*^*a*^
GAK	Gain	Gain	Normal	WT	Q61L	WT
HMV II	Gain	Normal	Loss	G469L	Q61K	WT

### Gene expression profile of PDX005 tumor tissue to CDK4/6 inhibitors

**Fig. 4:**
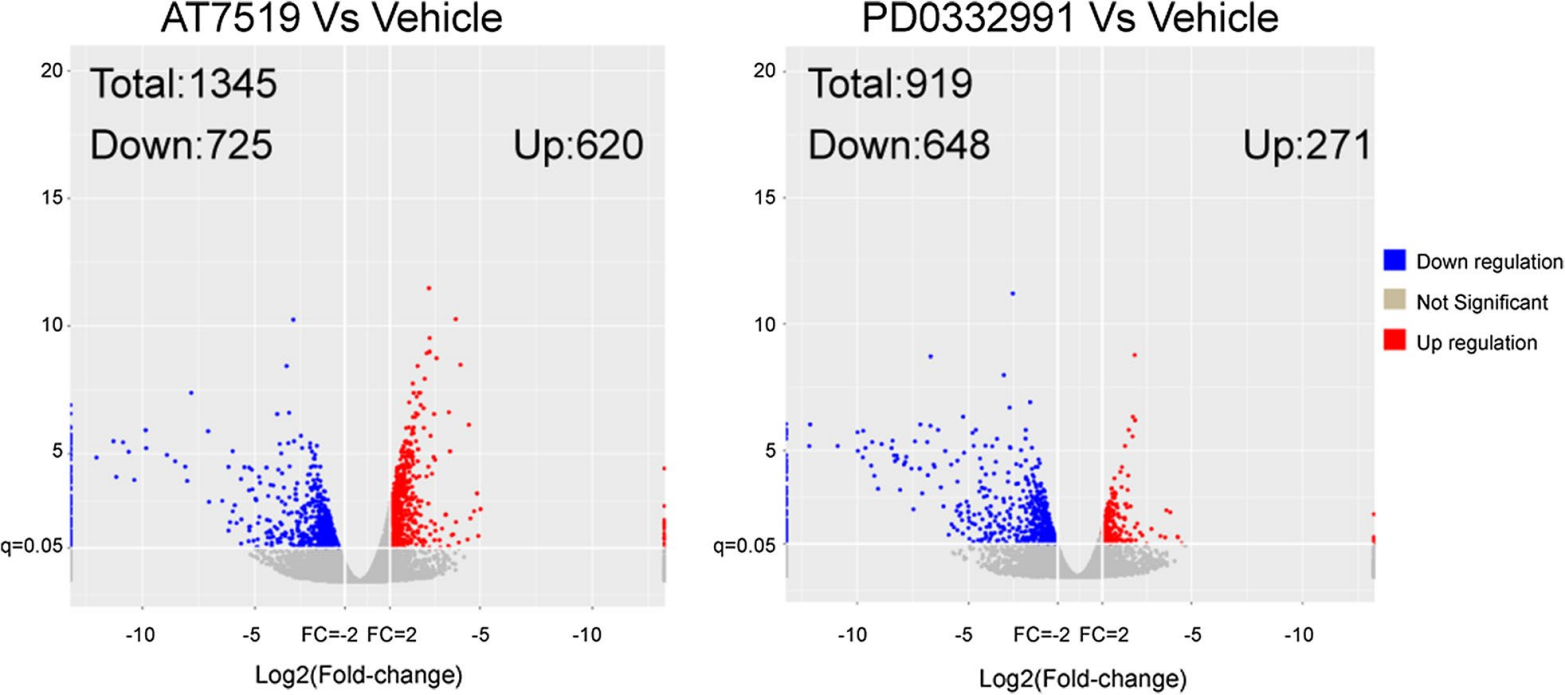
The volcanic map of PDX005 treated with CDK4/6 inhibitors

To identify the molecular alterations after CDK4/6 inhibitors treatment, mRNA sequencing was performed on PDX tumor cells isolated from three paired tissue samples. On average, 7.6 million reads (between 7.0 and 8.6 million per sample) were obtained using Illumina HiSeq 2500 platform of which 97% had high quality scores (≥ Q20). Compared with vehicle group, AT7519 treated PDX-005 tumors had 1345 DEGs (FC ≥ 2, adjusted P-values < 0.05), among which 725 genes were down-regulated and 620 genes were up-regulated. In PD0332991-treated tumors, a total of 919 DEGs (FC ≥ 2, adjusted P-values < 0.05) were observed, among which 648 genes were down-regulated and 271 genes were up-regulate. The volcanic map of down- and up-regulated genes was listed in Fig. 4.

**Fig. 5:**
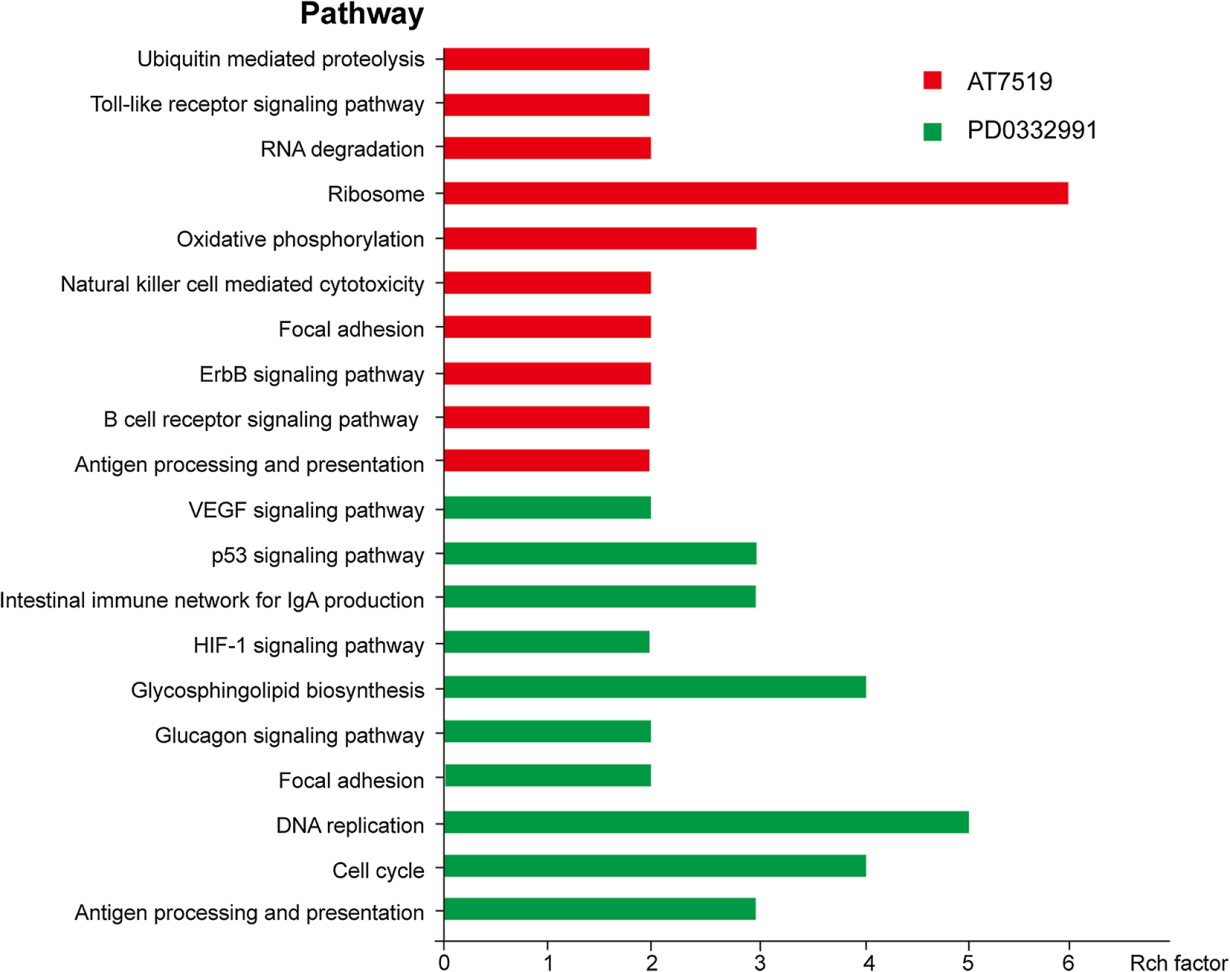
Analysis of the signal pathway regulated by CDK4/6 inhibitors by KEGG signal pathway

Functional annotation analysis of all DEGs utilizing: Profiler software revealed “Ribosome” (58 genes, P < 7.8E−24), “Oxidative phosphorylation” (32 genes, P < 4.8E−08), “Focal adhesion” (29 genes, P < 0.001) and “Antigen processing and presentation” (15 genes, P < 0.002) were the most enriched KEGG pathways for AT7519 vs vehicle. For PD0332991 vs vehicle group, “Cell cycle” (25 genes, P < 7.5E−9), “DNA replication” (9 genes, P-values 3.1E−05), “Focal adhesion” (25 genes, P < 5.6E−05) and “p53 signaling pathway” (11 genes, P < 0.0004) were the most enriched KEGG pathways. The top 10 pathways were showed in Fig. 5.

## Discussion

Melanoma is the fifth most common cancer in the Caucasian population [1]. CM is the major subtype melanoma in Caucasian population. Epidemiological statistics showed more than 5000 new cases of MM in China every year, and the incidence of MM is increasing [2]. Unlike CM, MM exhibits distinct biological and clinical features. Targeted therapies, such as BRAF inhibitors and c-KIT inhibitors, have greatly revolutionized the treatment of CM. However, the effect of such targeted therapy on the treatment for MM remained obscure due to following reasons: 1, the number of MM patients included in clinical trials is very limited. 2, the mutation rates of the BRAF and c-KIT genes were only about 13.9 and 9.6%, respectively, in MM patients [18, 19]. Thus, potential targets should be identified for MM patients for developing new and effective targeted therapies.

Copy number variation of CDK signaling pathway genes plays an important role in many tumors. Reducing the activity of CDK signaling pathway can significantly inhibit the growth of tumor. CDK4/6 and CCND1 genes were amplified while CDKN2A, CDKN2B and CDKN1B were deleted in primary and metastatic breast cancers [20]. In patients with type 1 neurofibromatosis associated with breast cancer, the expansion of CDK4 copy number may increase the expression of the NF1 gene and then up-regulate the expression of Her2 gene in breast cancer cells [21]. CDK4 is amplified and its protein expression is increased in esophageal squamous cell carcinoma (ESCC), and both of them are associated with the poor survival of ESCC. CDK4 silencing or PD0332991 treatment significantly inhibits the proliferation of esophageal cancer cells [22]. Similar results are observed that CDK4/6 inhibitors suppress the proliferation of thyroid cancer cells [23]. In addition, research for malignant glioma, neuroblastoma and malignant peripheral nerve sheath tumors demonstrated that CDK4 copy number was amplified and was associated with tumor prognosis [23–25]. Moreover, our previous study of 514 cases of acral melanoma showed that 87% of patients had at least one copy number variant of the CDK4 signaling pathway genes. Cdk4 gain, Ccnd1 gain and the combination of Cdk4 gain and Ccnd1 gain were associated with the poor prognosis of acral melanoma, respectively [16]. In this study, copy number amplification of CDK4 and CCND1 genes and copy number deletion of CDKN2A gene were observed in most MM cases, suggesting that CDK4 signaling pathway aberrations maybe the driver for MM. Further analysis showed that CNV was significantly associated with the TNM stage in MM. However, the aberration of CDK4 signaling pathway genes was not significantly associated with the OS of MM patients. Therefore, it is necessary to expand the sample for further verification. Richard et al. found that CDKN2A copy number loss was a frequent event in patients with CM, and the prognosis is worse in patients with increased Ccnd1 copy number and Cdk4 copy number. However, after stratification of the status of NRAS and Braf mutations in patients, copy number variation in the CDK4 signaling pathway genes was not significantly associated with the survival of CM [26]. This is similar to our results because the mutation rates of NRAS and Braf is extremely low in MM patients, and the patients analyzed tend to be independent of NRAS and BRAF mutation.

Previous studies have shown that CDK inhibitors can inhibit the growth of a variety of tumors. CDK4/6 inhibitor Palbociclib combined with mTOR inhibitor MLN0128 suppresses the proliferation of ER negative breast cancer cells and Glioma [12, 27]. Similarly, triple-negative breast cancers with MT4-MMP, EGFR and RB-positive are sensitive to Erlotinib in combination with Palbociclib in PDX model [28]. Our previous study found that both CDK inhibitors and specific CDK4/6 inhibitors effectively inhibited the tumor growth in PDX models of acral melanoma containing CDK4 pathway aberration [16]. Zhou et al. found that CDK4 gene was amplified in 65 MM patients and Palbociclib, an inhibitor of CDK4/6, effectively blunted the tumors harboring Cdk4 copy number gain in a PDX model [29]. Here, we found that both AT7519, a pan CDK inhibitor, and PD0332991, a selective CDK4/6 inhibitor, significantly inhibited the proliferation of MM cell lines in vitro. In vivo, both inhibitors obviously reduced the growth of tumors which harbored abnormal CDK4 signaling pathway in PDX models. However, tumors with normal CDK4 signaling pathway exhibited minimal sensitivity to both inhibitors. These results suggest that CDK4 aberration is an indicator for CDK4/6 inhibitors applied in MM. We also found in clinic that Palbociclib, an inhibitor of CDK4/6, can prolong the survival of MM patients with copy number variations of CDK4 pathway [30]. Therefore, CDK4 pathway genes copy number variation is a therapeutic target for MM.

To explore the potential molecular mechanisms, we subjected a PDX model (PDX-005) containing Cdk4 gain + Ccnd1 gain + P16^INK4a^ loss to RNA sequencing. Analysis of GO and KEGG revealed significant changes in several signaling pathways, including cell cycle and immunity. It is worth noting that in the enrichment analysis of the biological process, cellular component, molecular function and KEGG Pathway, the immune-related signaling pathways were significantly altered during the four above-mentioned analyses. These results suggest that CDK4/6 inhibitors may play an important role in the development of MM by regulating the cellular immune signaling pathway. Alterations in immune-related signaling pathways suggest that CDK4/6 inhibitors may affect the cellular immune system, thus affecting the efficacy of immunotherapies to tumor. Immunotherapy, including PD-1 antibody, has revolutionized the treatment of many tumors. In breast cancer, Shom et al. found that CDK4/6 inhibitors not only induce tumor cell cycle arrest, but also promote anti-tumor immunity in two main way: 1, CDK4/6 inhibitors alter tumor cell expression of endogenous retroviral components, hence increasing intracellular levels of double-stranded RNA. This in turn stimulates production of type III interferons and therefore enhances tumor antigen presentation. 2, CDK4/6 inhibitors markedly check the proliferation of regulatory T cells [31]. In general, the above-mentioned results suggest that CDK4/6 inhibitors are common in inducing antitumor immunity.

## Conclusion

In this study, we found that the copy number of the components of CDK4 signaling pathway is altered in MM. Inhibition of CDK signaling pathway and CDK4 signaling pathway alone obviously suppresses the proliferation of MM cells and the tumor growth in PDX models harboring CDK4 pathway abnormity. This study provides theoretical basis for targeting CDK4 pathway in MM.

## Additional file

**Additional file 1: Table S1.** Correlation of CDK4 pathway aberrations to treatment groups.

## Data Availability

All the data and materials supporting the conclusion were included in the main paper.

## Abbreviations

AM: acral melanoma
bDNA: branched DNA
CM: cutaneous melanoma
CNV: copy number variation
FDR: false discovery rate
FFPE: Formalin-Fixed, Paraffin-Embedded Tissues
FPKM: Fragments Per Kilobase of exon model per Million mapped read
GO: gene ontology
KEGG: Kyoto Encyclopedia of Genes and Genomes
MM: mucosal melanoma
NOD/SCID: non-obese diabetic and severe combined immunodeficiency
OS: overall survival
PDX: patient-derived xenograft model
QGP: QuantiGenePlex DNA assay
