# Correction to: Frequent genetic aberrations in the cell cycle related genes in mucosal melanoma indicate the potential for targeted therapy

**DOI:** 10.1186/s12967-019-2106-x

**Published:** 2019-11-05

**Authors:** Longwen Xu, Zhiyuan Cheng, Chuanliang Cui, Xiaowen Wu, Huan Yu, Jun Guo, Yan Kong

**Affiliations:** Key Laboratory of Carcinogenesis and Translational Research (Ministry of Education), Department of Renal Cancer and Melanoma, Peking University Cancer Hospital & Institute, 52 Fucheng Road, Beijing, 100142 China

## Correction to: J Transl Med (2019) 17:245 10.1186/s12967-019-1987-z

Following publication of the original article [1], the authors reported errors in Figures 2, 3 and Figure 3 ‘continued’.

1. In Figure 2b and 2f of PDX2 model, duplicated pictures of tumors have been used.
2. In Figure 3 of H&E staining of PDX-004, duplicated pictures have been used. Moreover, the description of the second PDX-001 was not correct in Figure 3.
3. In Figure 3 ‘continued’ of H&E staining, duplicated pictures have been used in all PDX groups. Moreover, the part labels in Figure 3 ‘continued’ were not correct.

In this Correction the corrected version of Figs. 2, 3 and Fig. 3 ‘continued’ are shown.

**Fig. 2:**
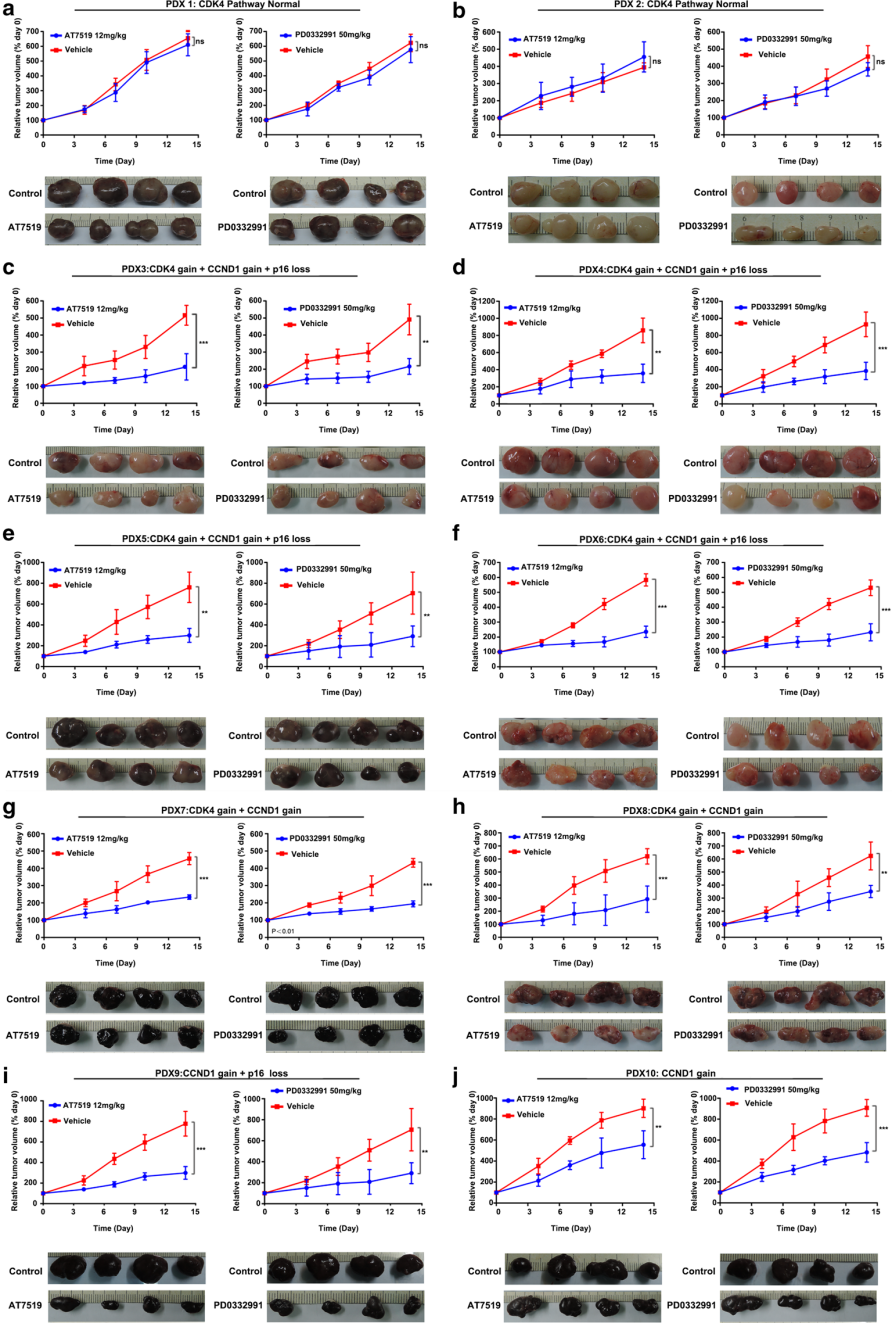
Sensitivity of PDX models containing CDK4 aberrations to CDK4/6 inhibitors in vivo. When the tumor size reached approximately 600 mm^3^, mice (n = 4 per group) were treated with buffer control or inhibitors daily. Tumor volume was evaluated as % of the tumor volume on day 0 and presented as mean ± SD. The comparison of the growth curves was done with the repeated measure variance analysis. *ns* no significances; **P < 0.01; ***P < 0.001

**Fig. 3:**
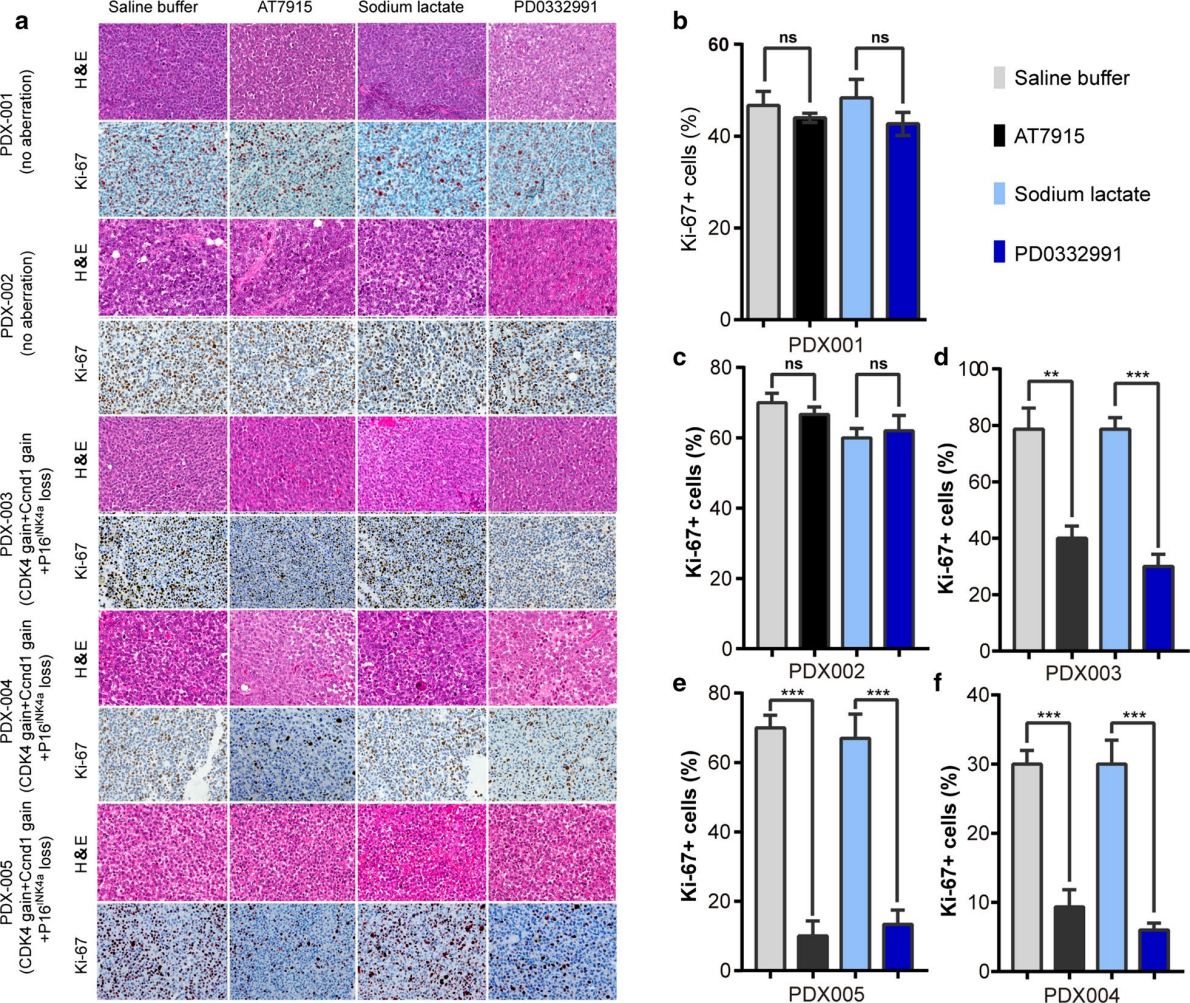

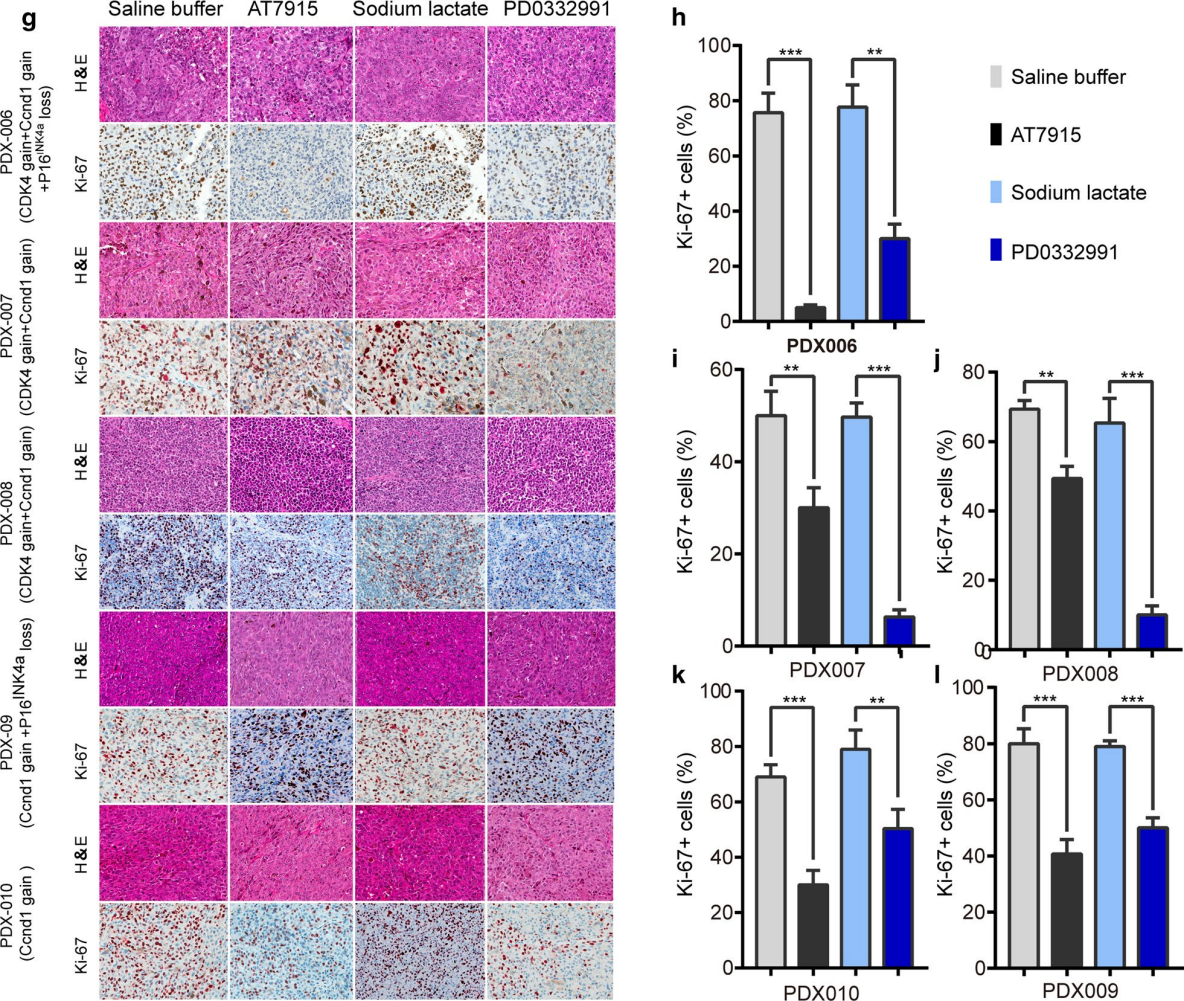
Proliferation index of mucosal melanoma cells from PDX models containing CDK4 aberrations after CDK4/6 inhibitors treatments. On day 14 of treatments, the tumor nodules were excised and examined by H&E staining and immunohistochemical staining (for Ki-67). The sections were evaluated under microscope, and typical staining was photographed (**a**). The Ki-67 + cells under 5 random fields were counted. Bar = 20 μm. The results of Ki-67 + cells (**b**–**f**) were presented as mean ± SD of three sections. *ns* no significances; *P < 0.05; **P < 0.01; ***P < 0.001
