# Supplementary material for: Frequent genetic aberrations in the cell cycle related genes in mucosal melanoma indicate the potential for targeted therapy
Source: J Transl Med. 2019 Jul 29;17:245. doi: 10.1186/s12967-019-1987-z (PMC6664769; doi:10.1186/s12967-019-1987-z)
Supplement: Supplementary file 1 — Additional file 1: Table S1. Correlation of CDK4 pathway aberrations to treatment groups. [file 12967_2019_1987_MOESM1_ESM.docx]

| **Clinicopathologic factor** |  | **CDK4 aberration** | | |  | **CCND1 aberration** | | | |
| --- | --- | --- | --- | --- | --- | --- | --- | --- | --- |
|  |  | **Gain** | **Normal** | **P value** |  | **Gain** | **Loss** | **Normal** | **P value** |
| Treatment^b^ |  |  |  | **0.003** |  |  |  |  | 0.118 |
| 1 |  | 77 (77.0) | 61 (55.5) |  |  | 42 (71.2) | 22 (57.9) | 75 (64.7) |  |
| 2 |  | 7 (7.0) | 17 (15.5) |  |  | 3 (5.1) | 4 (10.5) | 17 (14.7) |  |
| 3 |  | 7 (7.0) | 13 (11.8) |  |  | 6 (10.2) | 5 (13.2) | 10 (8.6) |  |
| 4 |  | 0 (0.0) | 3 (2.7) |  |  | 0 (1.0) | 0 (0.0) | 3 (2.6) |  |
| 5 |  | 4 (4.0) | 2 (1.8) |  |  | 2 (3.4) | 1 (2.6) | 3 (2.6) |  |
| 6 |  | 1 (1.0) | 9 (8.2) |  |  | 6 (10.2) | 2 (5.3) | 3 (2.6) |  |
| 7 |  | 4 (4.0) | 5 (4.5) |  |  | 0 (0.0) | 4 (10.2) | 5 (4.3) |  |

**Table S1**. **Correlation of CDK4 pathway aberrations to treatment groups**

**Table S1 continued**

| **Clinicopathologic factor** | **P16^INK4a^ aberration** | | |  | **Overall aberration (≥ 1 CNV)** | | |
| --- | --- | --- | --- | --- | --- | --- | --- |
|  | **Loss** | **Normal** | **P value** |  | **Yes** | **No** | **P value** |
| Treatment^b^ |  |  | 0.938 |  |  |  | 0.184 |
| 1 | 80 (65.0) | 59 (66.3) |  |  | 115 (66.1) | 24 (61.5) |  |
| 2 | 13 (10.6) | 11 (12.4) |  |  | 18 (10.3) | 6 (15.4) |  |
| 3 | 11 (8.9) | 9 (10.1) |  |  | 14 (8.0) | 7 (17.9) |  |
| 4 | 2 (1.6) | 1 (1.1) |  |  | 2 (1.1) | 1 (2.6) |  |
| 5 | 3 (2.4) | 3 (3.4) |  |  | 6 (3.4) | 0 (0.0) |  |
| 6 | 8 (6.5) | 3 (3.4) |  |  | 11 (6.3) | 0 (0.0) |  |
| 7 | 6 (4.9) | 3 (3.4) |  |  | 8 (4.6) | 1 (2.6) |  |

**Table S1 continued**

| **Clinicopathologic factor** | **CDK4 gain + CCND1 gain** | | |  | **CDK4 gain + P16^INK4a^ loss** | | |
| --- | --- | --- | --- | --- | --- | --- | --- |
|  | **Positive** | **Negative** | **P value** |  | **Positive** | **Negative** | **P value** |
| Treatment^b^ |  |  | 0.346 |  |  |  | 0.416 |
| 1 | 24 (80.0) | 114 (63.3) |  |  | 50 (75.8) | 88 (61.1) |  |
| 2 | 1 (3.3) | 23 (12.8) |  |  | 5 (7.6) | 19 (13.2) |  |
| 3 | 4 (13.3) | 16 (8.9) |  |  | 5 (7.6) | 15 (10.4) |  |
| 4 | 0 (0.0) | 3 (1.7) |  |  | 0 (0.0) | 3 (2.1) |  |
| 5 | 1 (3.3) | 5 (2.8) |  |  | 2 (3.0) | 4 (2.8) |  |
| 6 | 0 (0.0) | 10 (5.5) |  |  | 1 (1.5) | 9 (6.2) |  |
| 7 | 0 (0.0) | 9 (5.0) |  |  | 3 (4.5) | 6 (4.2) |  |

**Table S1 continued**

| **Clinicopathologic factor** | **P16^INK4a^ loss + CCND1 gain** | | |  | **CDK4 gain + P16^INK4a^ loss + CCND1 gain** | | |
| --- | --- | --- | --- | --- | --- | --- | --- |
|  | **Positive** | **Negative** | **P value** |  | **Positive** | **Negative** | **P value** |
| Treatment^b^ |  |  | 0.652 |  |  |  | 0.491 |
| 1 | 24 (72.7) | 115 (64.2) |  |  | 14 (82.4) | 125 (63.8) |  |
| 2 | 2 (6.1) | 22 (12.3) |  |  | 0 (0.0) | 24 (12.2) |  |
| 3 | 3 (9.1) | 17 (9.5) |  |  | 2 (11.8) | 19 (9.7) |  |
| 4 | 0 (0.0) | 3 (1.7) |  |  | 0 (0.0) | 3 (1.5) |  |
| 5 | 1 (3.0) | 5 (2.8) |  |  | 1 (5.9) | 5 (2.6) |  |
| 6 | 3 (9.1) | 8 (4.5) |  |  | 0 (0.0) | 11 (5.6) |  |
| 7 | 0 (0.0) | 9 (5.0) |  |  | 0 (0.0) | 9 (4.6) |  |

b. The treatment options include: 1. Dacarbazine or temozolomide +cisplatin +recombinant human endostatin；2. High-dose IFN-a2b；3. paclitaxel or paclitaxel albumin + carboplatin + bevacizumab or recombinant human endostatin; 4. Temozolomide+ Sorafenib+ bevacizumab;5.Targeted therapy (such as: BRAF inhibitor，C-kit inhibitor or PD1 inhibitor); 6.No treatment; 7.Recieve various treatments without according guidance.
